# Acculturation Experiences and Preterm Birth in Berlin: Does Acculturative Stress Contribute to Preterm Birth?

**DOI:** 10.1007/s10903-023-01480-7

**Published:** 2023-04-20

**Authors:** Marlene Lee, Anna Pöhlmann, Michael Abou-Dakn, Matthias David

**Affiliations:** 1 Department of Gynecology, Charité Universitätsmedizin Berlin, Corporate Member of Freie Universität Berlin and Humboldt Universität Zu Berlin, Campus Virchow Klinikum, Augustenburger Platz 1, 13353 Berlin, Germany; 2 Institute of Biometry and Clinical Epidemiology, Charité Universitätsmedizin Berlin, Corporate Member of Freie Universität Berlin and Humboldt Universität Zu Berlin, Campus Mitte, Charitéplatz 1, 10117 Berlin, Germany; 3 Department of Gynecology and Obstetrics, St. Joseph Krankenhaus Berlin Tempelhof, Wüsthoffstraße 15, 112102 Berlin, Germany

**Keywords:** Acculturation, Acculturative stress, Preterm birth, Perinatal outcome

## Abstract

Acculturation and acculturative stress are potential risk factors for adverse perinatal outcomes. This study investigates whether and how acculturative stress affects preterm birth (PTB) in a sample of migrant women in Berlin. We interviewed 955 women who recently gave birth using standardized questionnaires (Frankfurt Acculturation Scale and Acculturative Stress Index). Multivariable logistic regression analyses assessed the effects of acculturation and acculturative stress on PTB. Women with migrant backgrounds did not have significantly higher PTB rates than German natives. First-generation migrants experienced higher acculturative stress levels than second-generation migrants, 38.8% vs. 13.2%. Acculturative stress could not be identified as a risk factor for PTB in our sample. These results need to be considered in the context of an international city and the wide use of antenatal care services in our population, which could be responsible for similarly good perinatal outcomes and highlights the potential of good access to perinatal care for vulnerable groups.

**Supplementary Information:**

The online version contains supplementary material available at 10.1007/s10903-023-01480-7.

## Background

Over the past five decades, There has been an increase in international migration [1]. In Germany, in 2020, about 21.9 million people, making up around a quarter of the population, had a migrant background [2]. In the global context, a migrant is generally considered a person who resides in a foreign country than the country of which they are a national or citizen, irrespective of the cause or means of migration [3]. A second-generation migrant is a person who resides in a country where at least one of their parents entered as a migrant [3, 4]. The most common reasons for emigrating are work, education, or family. Only a relatively small percentage of migrants are forced to leave their home countries due to conflict, violence, or natural disasters [1].

Nevertheless, the migration experience presents a stressful life event, no matter the reasons for migration and whether these are voluntary or involuntary [5]. Many international studies have shown disparities in perinatal outcomes between migrants and receiving-country nationals. One main observation has emerged, namely more favorable perinatal outcomes of foreign-born migrants compared to natives, despite racial and socioeconomic disadvantages summarized as the "epidemiologic paradox"[6]. However, these favorable outcomes have decreased over time spent in the receiving country and migrant generations, resulting in less favorable outcomes for second-generation migrants compared to first-generation migrants.  This effect has been primarily attributed to acculturation [6, 7].

### Theoretical Framework

The concept of acculturation describes social and cultural changes resulting from the interaction between two cultures and can be associated with significant life events, which can present stressors [8–10]. The stress responses rooted in the acculturation experience are known as “acculturative stress” [8]. The acculturative stress model proposed by Berry suggests that potential stressors might be mitigated, depending on personal factors, such as age, gender, and coping mechanisms [8]. Based on the reasons for migration and the available coping mechanisms, adjustments to the receiving culture may cause acculturative stress, which occurs when the distressing migration experiences outweigh the moderating factors [8, 11].

Perinatal outcomes, particularly preterm birth (PTB), are a significant cause of infant morbidity and mortality, contributing largely to rising healthcare costs, and are an essential indicator of the health status of a society [12, 13]. Previous studies have identified acculturative stress as a factor for higher PTB rates of second-generation migrants compared to foreign-born migrants [7, 14]. In Germany, the effect of acculturation on perinatal health outcomes has been explored by David et al. [15, 16]. Their data suggests lower rates of PTB in first-generation Turkish and Lebanese women compared to non-migrants [16]. The previously described phenomenon of decreased favorable perinatal health outcomes of migrants over time spent in the receiving country and second-generation migrants have been attributed to acculturation [7]. However, there remains a need to study the potential effect of acculturative stress on perinatal outcomes in migrant women in Germany. This study explores the effects of acculturation and acculturative stress on PTB in migrant women in Berlin. The following research questions will be explored: 1) Is there a difference in the PTB rate of migrants and German natives? 2) Do high acculturation and acculturative stress levels lead to more PTB in women with a migrant background?

## Methodology

### Participants and Data Collection

A prospective single-center study at St. Joseph Krankenhaus Berlin Tempelhof, a major perinatal center in Berlin, Germany, was performed from May 2018 until December 2020. A random sample of women who had recently given birth (first-sixth day postpartum) and were admitted to the maternity ward was approached for participation. The inclusion criteria were: age ≥ 18 years, live birth of a child ≥ 24 weeks of gestation, in-hospital stay, and sufficient German language knowledge. Exclusion criteria were emergencies, communication difficulties, and declining participation in the study. Patients were asked to participate in a standardized in-person interview, which was administered by five previously trained interviewers. Patients who did not want to participate in an interview due to personal preference had the choice to complete the questionnaires by themselves. The questionnaires were administered in German.

### Questionnaires and Measures

The standardized questionnaires were comprised of four parts: 23 questions on socioeconomic data, two questions on stress level during pregnancy, 27 questions on migration and acculturation, as well as 21 questions on acculturative stress. The acculturation level was measured by the "Frankfurter Akkulturationsskala" (FRAKK), i.e., the "Frankfurt acculturation scale” [17]. The sum of two scales was calculated: "orientation to the culture of origin" and "orientation to the culture of the receiving country" and then summarized into an acculturation index, which mirrors the individual's overall dimension of acculturation [17]. The values of the FRAKK were then categorized into the following frequency quartiles: low ≤ 25th, medium > 25th– < 75th, and high ≥ 75th percentile.

The acculturative stress level was measured using the "Acculturative Stress-Index" (ASI), which was first introduced by Nicassio [18] and adapted for Germany by Foroutan [19]. One point per item is given to the answer that represents a stressor. The sum was determined and grouped into the same frequency quartiles as the FRAKK explained above, resulting in three ASI groups: low, medium, and high.

Additionally, antenatal and perinatal data, routinely reported for quality assurance purposes, were collected from patient records and merged with the questionnaire data. Migration background was defined according to the specification of the German Census Bureau as follows: a) born in a foreign country (first-generation migrant), b) mother and/or father was born in a foreign country (second-generation migrant) [2]. Data were compared between first- and second-generation migrants and non-migrants. Only singleton pregnancies were considered in the final analyses because of the differences in perceived stress levels and experiences linked to twin pregnancies and births. The primary outcome variable was preterm birth (< 37 + 0 weeks of gestation). Other variables were sociodemographic characteristics and pregnancy-related data.

### Statistical Analyses

Data were analyzed using SPSS version 28.0 (Armonk, NY: IBM Corp, USA) and Graph Pad Prism version 9.0 (La Jolla, CA, USA). Descriptive analyses were performed to compare relevant perinatal and maternal health data between first- and second-generation migrants and non-migrants. Chi-squared and one-way ANOVA tests were used to assess differences in the birth outcomes between first- and second-generation migrants and women with no migrant background.

#### Multivariable Logistic Regression Analyses

Multivariable logistic regression analyses were performed to assess the effects of acculturation and acculturative stress on preterm birth.

Two separate logistic regression analyses with the dependent variable preterm birth (< 37 + 0 weeks of gestation) (yes/no) with the following categorical variables were performed: FRAKK (low, medium, high) or ASI (low, medium, high), respectively. The bivariate variables risk factor for preterm birth (yes/no), and parity (nulliparous vs. multiparous) were also added to the models. We adjusted for the potential confounders age ≥ 35 years [20–23], BMI > 30 [16, 23–25], and low education level [16, 20]. No adjustments were made for multiple testing. Spearman correlation analyses were performed to assess the potential multicollinearity.

#### Selection of Variables

The variables for the multivariable log. regression analyses were chosen based on a literature review. The most important risk factors for PTB, which were also available in our data, are listed in Table 1. All risk factors were summarized into one variable, the occurrence of at least one risk factor for PTB (yes/no). Since the risk for PTB is highest in nulliparous compared to multiparous women [26], this variable was also included in the model: parity (nulliparous vs. multiparous). The correlations between FRAKK/ASI and migrant generation were very high: (Sr = 889; p-value < 0.001) for FRAKK and (Sr = 938; p-value < 0.001) for ASI, respectively. Therefore, to prevent multicollinearity, we did not include migrant generation in the models.

**Table 1:** Risk factors for preterm birth

Variable	Total	No migrant background	1st migrant generation	2nd migrant generation
	n = 896^a^	n = 454 (50.7%^a^	n = 282 (31.5%)^a^	n = 160 (17.9%)^a^
*Risk factor for PTB*				
1 risk factor	n = 142 (15.8%)	n = 72 (15.8%)	n = 38 (13.5%)	n = 32 (20%)
2 risk factors	n = 14 (1.6%)	n = 6 (1.3%)	n = 6 (2.13%)	n = 2 (1.3%)
*Short cervical length*				
< 25 mm	n = 19 (2.1%)	12 (2.6%)	2 (0.7%)	5 (3.1%)
*Bleeding in late pregnancy*				
> 28 weeks	n = 38 (4.2%)	19 (4.2%)	15 (5.3%)	4 (2.5%)
*Interpregnancy latency*				
< 12 months	n = 33 (3.7%)	17 (3.7%)	7 (2.5%)	9 (5.6%)
Preeclampisa	n = 27 (3%)	15 (3.3%)	5 (1.8%)	7 (4.4%)
Prior preterm delivery	n = 26 (2.9%)	15 (3.3%)	9 (3.2%)	2 (1.3%)
Conization	n = 4 (0.4%)	2 (0.4%)	2 (0.7%)	0
Smoking	n = 18 (2%)	4 (0.9%)	5 (1.8%)	9 (5.6%)

^a^No missings

### Handling of Missing Data

Only missing values in the FRAKK and ASI questionnaires were replaced, and up to three missings were replaced by imputation using the most frequent values. The data were excluded from analyses in the case of more than three missings. This way, 47 cases for FRAKK and 70 for ASI were excluded from the analyses. Missing data for other variables were not considered.

### Ethical Approval

Ethical approval for this study was obtained by the Ethics Committee of the Aerztekammer Berlin on 04/24/2018, registration number: Eth-09/18. Recommendations for good clinical practice and the data protection policy of Charité—Universitätsmedizin Berlin and the Berlin data protection act were followed.

## Results

### Patient Characteristics

**Fig. 1:**
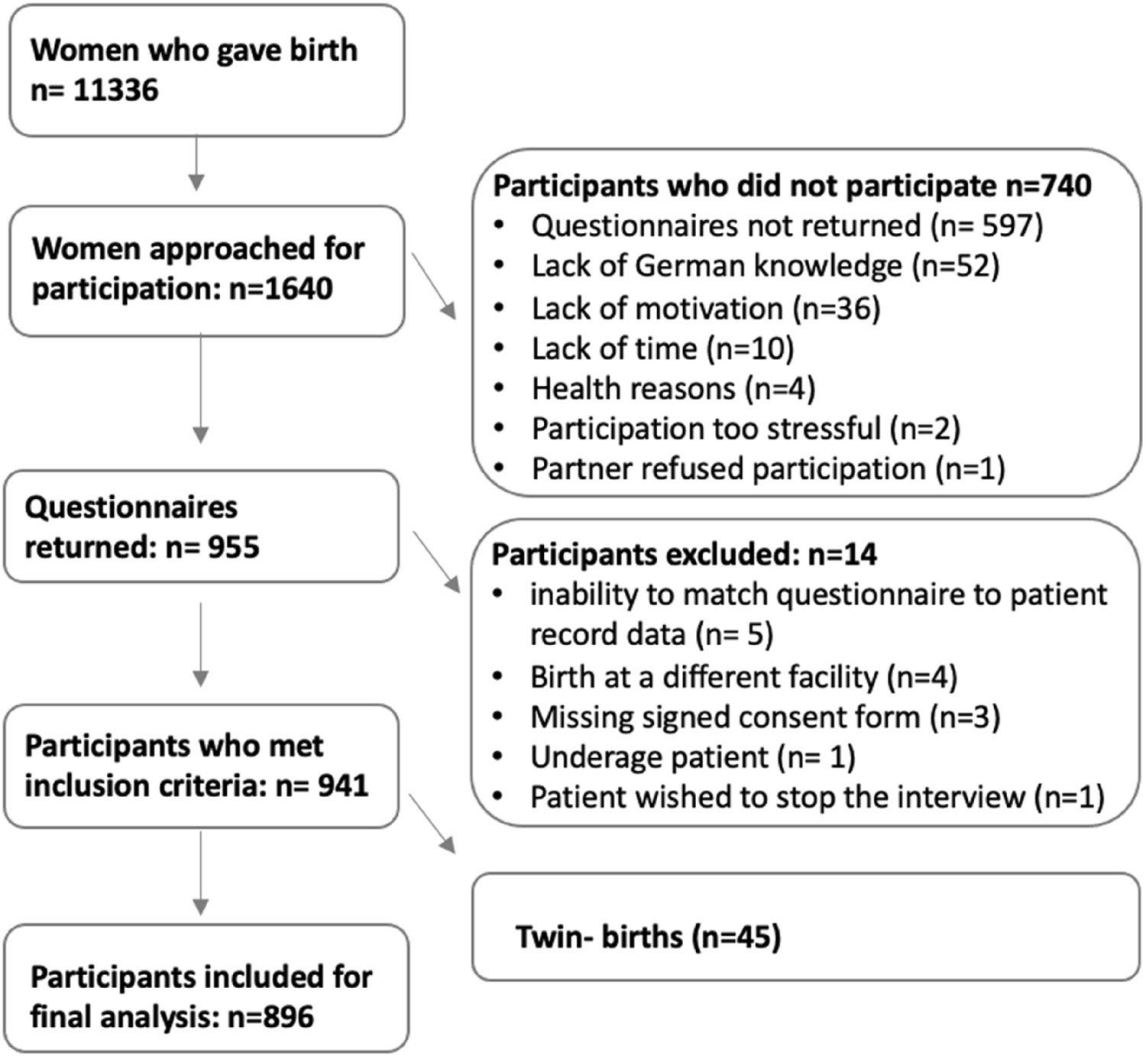
patient enrollment

During data collection, 11,336 women gave birth at the study center. A random sample of 1640 women (14.5%) was approached, of whom 955 participated in the study (return-rate = 58.23%). After the exclusion process *(*Fig. 1*), 896* patients remained for final analyses. Of these, 282 were first-generation migrant women, 160 were second-generation migrant women and 454 women with no migrant background. Second-generation migrants were younger (mean age 30.47 years) compared to first-generation migrants (mean age 32.59 years) and non-migrants (mean age 32.57 years). There were fewer women with high education levels among second-generation migrants (68.8%) compared to 83.7% of first-generation and 88.1% of non-migrants. The rate of primiparous women was quite similar in all three groups: 62.6% in non-migrants, 59.4% in second-generation migrants, and 57.4% in first-generation migrants (Table 2).

**Table 2:** Sociodemographic characteristics

Variable	Total	No migrant background	1st migrant generation	2nd migrant generation
	n = 896	n = 454 (50.7%)	n = 282 (31.5%)	n = 160 (17.9%)
*Age (years)*				
Min–max. age	18–49	19–46	18–49	19–43
Mean age (SD)	32.57 (5.05)	33,28 (4.6)	32.59 (5.2)	30.47 (5.5)
*Education level*				
High school and higher	n = 746	400 (88.1%)	236 (83.7%)	110 (68.8%)
No education– secondary school	n = 148	52 (11.5%)	46 (16.3%)	50 (31.3%)
*Employment (yes)*	n = 724	409 (90%)	193 (68.4%)	122 (76.3%)
*Employment status*				
Employed	n = 649	378 (83.1%)	167 (59.4%)	104 (65%)
Self-employed	n = 44	22(4.8%)	16(5.7%)	6 (3.8%)
Student/apprentice	n = 54	15 (3.3%)	26 (9.3%)	13 (8.1%)
House wife	n = 84	16 (3.5%)	48 (17.1%)	20 (12.5%)
Medical leave	n = 45	16 (3.5%)	15 (5.3%)	14 (8.8%)
Other	n = 14	6 (1.3%)	7 (2.5%)	1 (0.6%)
*In a relationship*	n = 881	447 (98.2%)	276 (97.9%)	159 (99.4%)
*Family members in Berlin*	n = 535	296 (65.1%)	105 (37.5%)	134 (83.8%)
*Length of stay in Germany*				
< 5 years	–	–	51 (18.1%)	–
5 -14 years	–	–	133 (47.2%)	–
≥ 15 years	–	–	87 (30.9%)	–
*Good German language knowledge*	–	–	172 (61%)	–
*Reasons for migration*				
As a child with parents	–	–	57 (20.28%)	–
Moved to spouse/partner	–	–	71 (25.2%)	–
Work/education	–	–	105 (37.4%)	–
Political reasons	–	–	23 (8.19%)	–
German settler	–	–	7 (2.5%)	–
As a tourist	–	–	10 (3.6%)	–
Other reasons	–	–	8 (2.8%)	–

Of the 896 patients, 613 were born in Germany. The 283 women who were born abroad came from 76 different countries (Supplement Table 1). The most prevalent birthplaces were Poland (16.9%), Russia (5.3%), Syria, and Turkey (4.9%, respectively). Most of the women migrated for work or education (37.4%), followed by moving to their spouse/partner (25.2%) and moving as a child with their parents (20.28%). Only 8.19% of this population emigrated due to political reasons (Table 2).

### Acculturation and Acculturative Stress Levels

**Fig. 2:**
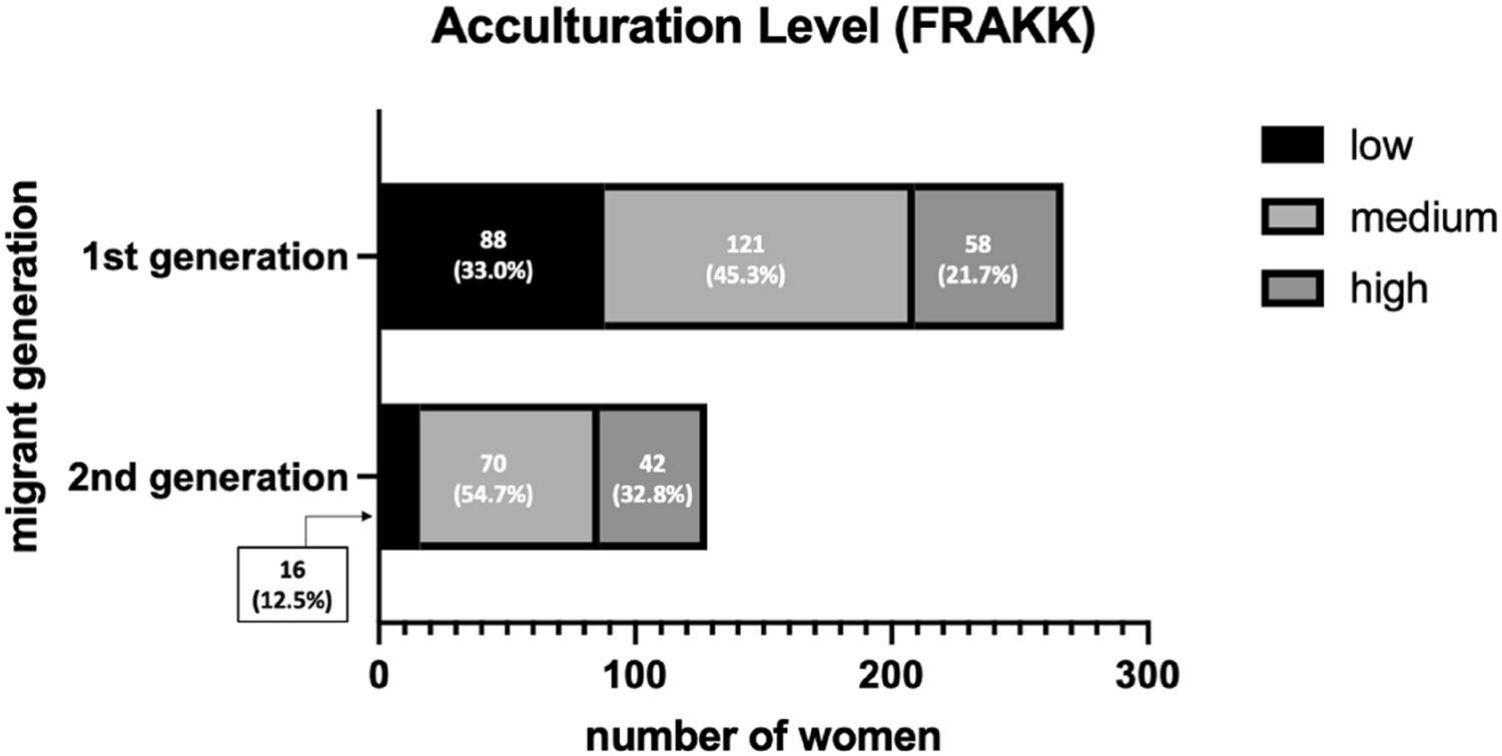
Acculturation levels according to migrant generation. n = 395, missings n = 47

**Fig. 3:**
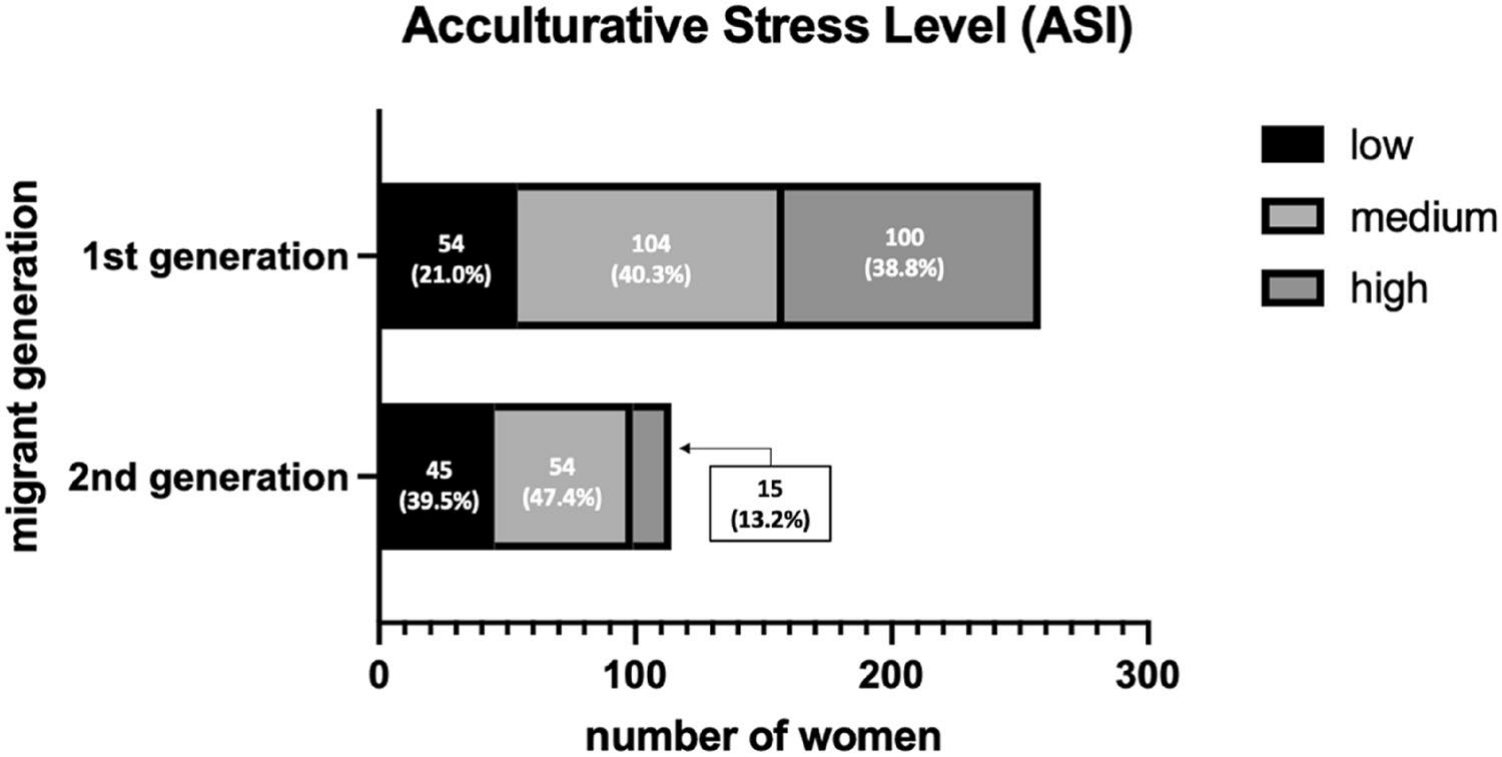
Acculturative stress levels according to migrant generation. n = 372, missings: n = 70

Of the 442 women with a migrant background, FRAKK scores were calculated for 395 (89.4%), and ASI scores were calculated for 372 (84.2%) women. More first-generation migrants had low FRAKK scores, 88 (33.0%), compared to second-generation migrants, 16 (12.5%). Medium FRAKK levels were similar in both groups: 121 (45.3%) in first-generation migrants, compared to 70 (54.7%) in second-generation migrants. Second-generation migrants had higher FRAKK scores, 42 (32.8%) compared to first-generation migrants, 58 (21.7%) (Fig. 2). ASI scores were higher in first-generation migrants: high for 100 (38.8%), medium 104 (40.3%), and low for 54 (21.0%), compared to second-generation migrants, where 15 (13.2%) had a high, 54 (47.4%) had a medium, and 45 (39.5%) a low ASI score (Fig. 3).

A Spearman correlation analysis was performed to determine whether there was a correlation between FRAKK and ASI. The analysis showed a *medium* negative correlation between acculturation level (FRAKK) and acculturative stress level (ASI) (Sr = -0.450; p-value < 0.001), in the sense that women who were more acculturated experienced lower levels of acculturative stress.

### Birth Outcomes

The levels of PTB were higher in second-generation migrants (13.8%) than first-generation migrants (9.9%) and non-migrants (9%) (p = 0.233). The rates of low umbilical cord pH (< 7.10) were also higher in second-generation migrants (6.9%) compared to first-generation migrants (3.9%) and non-migrants (3.5%), respectively (p = 0.187). The infant birth weight was marginally lower in second-generation migrants (mean 3327 g) than in the other two groups (3398 g in first-generation and 3410 g in non-migrants) (p = 0.284) (Table 3).

**Table 3:** Pregnancy-related characteristics and birth outcomes

Variable	Total	No migrant background	1st migrant generation	2nd migrant generation	p-value
	n = 896	n = 454 (50.7%)	n = 282 (31.5%)	n = 160 (17.9%)	
*Birth mode*					0.001^a^
Vaginal birth	n = 504	247 (54.4%)	156 (55.3%)	101 (63.1%)	
Assisted vaginal birth					
Primary cesarean					
Emergency cesarean	n = 92 n = 130 n = 170	56 (12.3%) 57 (12.5%) 94 (20.7%)	25 (8.9%) 59 (20.9%) 42 (14.9%)	11 (6.9%) 14 (8.8%) 34 (21.25%)	
*Antenatal care visits*					
> 10 visits	n = 684	363 (80%)	199 (70.6%)	122 (76.3%)	0.016^a^
*Early first ultrasound*					
< 9 weeks	n = 357	173 (38.1%)	117 (41.5%)	67 (41.9%)	0.558^a^
*Diabetes (yes)*	n = 134	63 (13.8%)	46 (16.3%)	25 (15.6%)	0.644^a^
*Induction of labor*	n = 153	74 (16.3%)	54 (19.2%)	25 (15.6%)	0.525^a^
*Epidural anesthesia*	n = 183	91 (20%)	47 (16.7%)	45 (28.1%)	0.016^a^
*Parity* = *1*	n = 162	284 (62.6%)	162 (57.4%)	95 (59.4%)	0.372^a^
*Parity* > *1*	n = 120	170 (37.4%)	120 (42.6%)	65 (40.6%)	
***Preterm birth*** ** < 37 weeks**	**n = 91**	**41 (9%)**	**28 (9.9%)**	**22 (13.8%)**	**0.233**^**a**^
*Birth weight (g)*					
Min.-max	995–5130	1200–5130	995–4830	1200–4740	0.264^b^
Mean (SD)	3391 (555,77)	3410 (552,71)	3398 (550,47)	3327 (572,35)	
*Low cord pH (*< *7.10)*	n = 38	16 (3.5%)	11 (3.9%)	11 (6.9%)	0.187^a^
*Low Apgar (*< *8) at 5 min*	n = 10	6 (1.3%)	4 (1.4%)	0	NA^c^
*Transfer to neonatal unit*	n = 123	54 (11.9%)	38 (13.5%)	31 (19.4%)	0.075^a^

^a^ Comparisons between the three groups were made using Chi^2^ test

^b^ Comparisons between the three groups were made using one-way ANOVA

^c^ No statistical test possible due to the small number of observations

p ≤ 0.05 was considered as statistically significant

### Risk Factors for Preterm Birth

In our sample, women had a maximum of two risk factors for PTB. At least one risk factor for PTB was present in 142 individuals (15.8%) of our population. 72 (15.8%) women with no migrant background, 38 (13.5%) women with 1st generation migrant background, and 32 (20%) women with 2nd generation migrant background had at least one risk factor for PTB. Bleeding in late pregnancy (< 28 weeks of gestation) was most common (4.2%), followed by short interpregnancy latency (< 12 months) and prior preterm delivery (2.9%). A history of conization was least frequent at only 0.4%.

### Effects of Acculturation and Acculturative Stress on the Preterm Birth Rate

We could not find a statistically significant difference in the PTB rates of German natives compared to women with a migrant background (p = 0.233) (Table 2).

Multivariable logistic regression analyses were performed to assess the effects of FRAKK and ASI on PTB. Tendencies of a higher risk of PTB in relation to high levels of acculturation and acculturative stress could be observed. However, there was no statistically significant association between the risk of experiencing PTB and high FRAKK- (p = 0.27) or high ASI-levels (p = 0.39) (Tables 4 and 5). Having at least one risk factor for PTB increased the risk of experiencing PTB by about fourfold in both models (OR = 4.34 for FRAKK and OR = 4.04 for FRAKK (p < 0001). No considerable difference in the models could be found before or after adjusting for the potential confounders age, BMI, and education level (Tables 4 and 5).

**Table 4:** Log. regression analysis: chance of preterm birth (acculturation level FRAKK)

	OR [95% CI]	p-value
Acculturation (FRAKK)		
Low	1.423 [0.624; 3.245]	0.401
Medium	1.659 [0.624; 4.411]	0.310
High	1.654 [0.674; 4.056]	0.272
*Ref.: no migrant background*		
Risk factor for PTB	4.336 [2.638; 7.125]	< 0.001*
*Ref.: no risk factor for PTB*		
Parity = 1	0.693 [0.419; 1.146]	0.153
*Ref.: parity* > *1*		
Age ≥ 35 years	0.957 [0.591; 1.644]	0.957
*Ref.:* < *35 years*		
BMI > 30	0.702[0.322; 1,527]	0.372
*Ref.: BMI* < *30*		
Education level low up to secondary school	0.604 [0.330; 1,103]	0.101
*Ref.: high school or higher*		
Constant	0.098	< 0.001
n = 820, missing = 76		
Nagelkerke R^2^ = 0.02		

*Ref.* reference, *OR* odds ratio, *95% CI * 95% confidence interval

*p ≤ 0.05 was considered as statistically significant

**Table 5:** Log. regression analysis: chance of preterm birth (acculturative stress level ASI)

	OR [95% CI]	p-value
*Acculturative stress (ASI)*		
Low	1.098 [0.528; 2.280]	0.803
Medium	0.686 [0.246; 1.914]	0.472
High	1.433 [0.629; 3.263]	0.392
*Ref.: no migrant background*		
Risk factor for PTB	4.043 [2.436; 6.710]	< 0.001*
*Ref.: no risk factor for PTB*		
Parity = 1	0.688 [0.413; 1.146]	0.151
*Ref.: parity* > *1*		
Age ≥ 35 years	1.083 [0.645; 1.819]	0.763
*Ref.:* < *35 years*		
BMI > 30	0.705 [0.324; 1.536]	0.379
*Ref.: BMI* < *30*		
Education level low up to secondary school	0.505 [0.275; 0.924]	0.027
*Ref.: high school or higher*		
Constant	0.132	< 0.001
n = 798, missings = 98		
Nagelkerke R^2^ = 0.108		

*Ref.* reference, *OR* odds ratio, *95% CI* 95% confidence interval

*p ≤ 0.05 was considered as statistically significant

## Discussion

In the present study, women with migrant backgrounds did not have a significantly higher PTB rate than German natives. Furthermore, we could not find a statistically significant association between acculturation or acculturative stress and PTB in our sample. These findings are consistent with some previous findings [27]. However, they are inconsistent with other results, which show an increase in adverse perinatal outcomes in foreign-born migrants compared to natives, summarized as the epidemiologic paradox [6, 12, 28, 29]. This phenomenon has primarily been attributed to acculturation and is widely studied in the Hispanic population in the United States [6, 7, 30, 31].

However, there remains a discussion about the applicability of the epidemiologic paradox across different migrant groups and the influence of ethnicity and race [6, 32–34].

Regarding ethnicity and race, according to the Centers for Disease Control and Prevention (CDC), in 2021, the PTB rate among African-American women (14.8%) was about 50% higher than among Hispanic (9.5%) and white women (10.2%) [34]. In a study by Acevedo-Garcia et al., there was an association of first-generation migrant status with low birth weight; the direction and strength of the association varied across different ethnic groups. We did not include questions on race/ethnicity in our questionnaires, which presents a limitation. Therefore, the role of racial or ethnic heterogeneity within immigrant groups needs to be addressed further in the context of adverse perinatal outcomes, particularly in a German-speaking country setting, as the migrants’ countries of origin differ from those of the United States [32, 33, 35].

In our population, second-generation migrants had a higher occurrence (20%), and first-generation migrants had a lower occurrence (13.5%) of risk factors for PTB compared to women with no migrant background (13.5%). These findings are consistent with the distribution of the PTB rate in our sample. There was a difference in the types of risk factors across the groups: smoking (5.6%) and a short birth interval (5.6%) occurred most frequently among second-generation migrants. These two factors are also linked to a lower socioeconomic background [36, 37], an individual risk factor for PTB, and a lower acculturation level [16, 25].

The present study's findings need to be considered in the context of the study population and setting. Berlin is an international city with a high density of medical care providers, including ante- and perinatal care facilities. Berlin is also a destination where many people immigrate for reasons such as work, study, and family, which may not cause a big psychosocial challenge to the individuals [1]. This is likely to be true in our population, with the most common reasons for migration being work, education, and family, making up 83% of the migration reasons. In contrast, only 8% emigrated due to political reasons. The acculturation experience is highly individual, and reasons for migration must be considered. They cannot readily be compared to another group previously studied, which may have been more vulnerable due to a different migration experience.

In previous studies, poor access to and quality of health care were identified as contributing factors in adverse perinatal health outcomes, and it has been shown that there is often an underutilization of health care services by immigrants, which can further contribute to poor perinatal outcomes [27, 38–40]. Additionally, lower socioeconomic background and education levels have been identified as contributing factors to poor birth outcomes [41, 42]. The majority of women in our study population came from a middle- to high-income socioeconomic background and had high levels of education (high-school degree or higher), 88% of women with no migrant background versus 84% of the first generation-, and 69% of second-generation migrants. Additionally, the women in our sample had a high rate of antenatal care visits (> 70%) and early first ultrasound examinations, which are measures of the access and quality of antenatal health care. The wide access and use of high-quality antenatal care services in our population could be responsible for similarly good perinatal outcomes in the three groups. This finding emphasizes the potential of low-barrier access and wide usage of high-quality antenatal care services as mitigating factors for adverse perinatal outcomes associated with acculturation.

### Strengths and Limitations

The strengths of this study are the rigorous design and the setting in a hospital located in an area with a high percentage of migrants in a big international city. Another considerable strength is the standardized method of data collection and standardized instruments to measure acculturation and acculturative stress rather than relying on proxy measures.

Furthermore, the comprehensive literature-based selection of variables for the multivariable log. regression models, including the most relevant risk factors for PTB with the highest relative risk or odds ratio, present a considerable strength [25, 41].

This study has important limitations, such as the relatively small sample size, particularly of second-generation migrants. In addition, our study population comes in different proportions from various countries of origin, so specific cultural aspects could not be adequately investigated. The necessity of sufficient German knowledge to complete the questionnaire is a significant limitation that could lead to the data loss of particularly vulnerable groups, as language is an essential factor in access to health services and health-related outcomes [30, 43]. Furthermore, we did not include questions on race/ethnicity, which have shown to affect the direction and strength of the epidemiologic paradox [33] and should be addressed in future research. The comprehensive inclusion of potential risk factors for PTB relies heavily on self-reporting and correct documentation from healthcare professionals. Therefore, the occurrence of risk factors may not be fully depicted in our sample.

Furthermore, the following bias need to be considered: imputation with most frequent values was used, which can lead to a distortion of the results. In order, to minimize this bias, a maximum of three missings were imputed for FRAKK- and ASI questionnaires and other missings were excluded from the analyses. To minimize social-desirability bias, one-on-one interviews were conducted whenever possible, and participants had the option of completing the questionnaire by themselves.

### New Contribution to the Literature and Outlook

To the best of our knowledge, this is the first study to examine the effects of acculturative stress on preterm birth and adverse perinatal outcomes in first- and second-generation migrants in Germany and German-speaking countries. In the present study population, no significant difference in PTB and other perinatal outcomes between women with migrant backgrounds and German natives could be found. However, the number of adverse events was generally low, i.e., PTB (10.1%) and low cord pH (4.2%).

In this study, acculturation and acculturative stress could not be identified as risk factors for PTB, which is in contrast to international findings. However, this is the first study to examine the effects of acculturative stress on PTB in a German-speaking country setting. In previous studies, a discrepancy in antenatal care could be observed as a risk factor for poor birth outcomes [27]. The highly standardized ante- and perinatal care in Germany, which could be observed in our sample, could present a mitigating factor to PTB rates, as potential risk factors for PTB can be detected and adressed early on.

A bigger sample size and questions including race and ethnicity to further analyze these effects is needed. The findings of this study cannot easily be generalized to other populations. Socioeconomic factors and factors related to the access and quality of health care, such as a more rural setting, need to be investigated further in the context of PTB and acculturation. Furthermore, multi-dimensional approaches to measure acculturation must be considered to depict this multifaceted phenomenon and link it more comprehensively to health outcomes. Nevertheless, the results of this study provide a first insight into the effects of acculturative stress on perinatal outcomes in a German-speaking country setting and highlight the potential of wide access to antenatal care services for vulnerable groups.

## Supplementary Information

Below is the link to the electronic supplementary material.

Supplementary file1 (DOCX 35 kb)

## Abbreviations

PTB: Preterm birth
FRAKK: Frankfurt Acculturation Scale
ASI: Acculturative Stress Index
ANC: Antenatal care
GW: Gestational week
BMI: Body mass index
OR: Odds ratio
95% CI: 95% Confidence interval
