# Supplementary material for: Acculturation Experiences and Preterm Birth in Berlin: Does Acculturative Stress Contribute to Preterm Birth?
Source: J Immigr Minor Health. 2023 Apr 20;25(4):765–74. doi: 10.1007/s10903-023-01480-7 (PMC10310617; doi:10.1007/s10903-023-01480-7)
Supplement: Supplementary file 1 — Supplementary file1 (DOCX 35 kb) [file 10903_2023_1480_MOESM1_ESM.docx]

**Supplement table 1: Birth countries of foreign-born women**

| Birth Country | Number of women (%)  *n=283* |
| --- | --- |
| Poland | 48 (16.9%) |
| Russia | 15 (5.3%) |
| Syria | 14 (4.9%) |
| Turkey | 14 (4.9%) |
| Italy | 11 (3.9%) |
| Spain | 11 (3.9%) |
| China | 10 (3.5%) |
| Lebanon | 9 (3.2%) |
| Ukraine | 9 (3.2%) |
| Austria | 6 (2.1%) |
| Brazil | 6 (2.1%) |
| Macedonia | 6 (2.1%) |
| Romania | 6 (2.1%) |
| Serbia | 6 (2.1%) |
| USA | 6 (2.1%) |
| Bulgaria | 5 (1.8%) |
| Greece | 5 (1.8%) |
| Iran | 5 (1.8%) |
| Bosnia | 4 (1.4%) |
| Afghanistan | 3 (1.1%) |
| Colombia | 3 (1.1%) |
| India | 3 (1.1%) |
| Kazakhstan | 3 (1.1%) |
| Kosovo | 3 (1.1%) |
| Moldova | 3 (1.1%) |
| Azerbaijan | 2 (0.7%) |
| Bangladesh | 2 (0.7%) |
| Belgium | 2 (0.7%) |
| Croatia | 2 (0.7%) |
| Czech Republic | 2 (0.7%) |
| France | 2 (0.7%) |
| Georgia | 2 (0.7%) |
| Indonesia | 2 (0.7%) |
| Iraq | 2 (0.7%) |
| Israel | 2 (0.7%) |
| Kuwait | 2 (0.7%) |
| Mongolia | 2 (0.7%) |
| Peru | 2 (0.7%) |
| Portugal | 2 (0.7%) |
| Slovakia | 2 (0.7%) |
| Sri Lanka | 2 (0.7%) |
| Switzerland | 2 (0.7%) |
| Tunisia | 2 (0.7%) |
| Algeria | 1 (0.4%) |
| Argentina | 1 (0.4%) |
| Armenia | 1 (0.4%) |
| Belarus | 1 (0.4%) |
| Bolivia | 1 (0.4%) |
| Cameroon | 1 (0.4%) |
| Canada | 1 (0.4%) |
| Chile | 1 (0.4%) |
| Cuba | 1 (0.4%) |
| Dominican Republic | 1 (0.4%) |
| Ecuador | 1 (0.4%) |
| Estonia | 1 (0.4%) |
| Guinea | 1 (0.4%) |
| Holland | 1 (0.4%) |
| Iceland | 1 (0.4%) |
| Japan | 1 (0.4%) |
| Jordan | 1 (0.4%) |
| Latvia | 1 (0.4%) |
| Mali | 1 (0.4%) |
| Mexico | 1 (0.4%) |
| Pakistan | 1 (0.4%) |
| Philippines | 1 (0.4%) |
| Senegal | 1 (0.4%) |
| Somalia | 1 (0.4%) |
| South Africa | 1 (0.4%) |
| Taiwan | 1 (0.4%) |
| Tajikistan | 1 (0.4%) |
| Thailand | 1 (0.4%) |
| UK | 1 (0.4%) |
| Vietnam | 1 (0.4%) |
